# Why inverse simulations overestimate optimal ankle exoskeleton assistance: the role of bi-articular coordination and joint stiffness

**DOI:** 10.1186/s12984-026-02106-3

**Published:** 2026-07-21

**Authors:** Israel Luis, Elena M. Gutierrez-Farewik, Maarten Afschrift

**Affiliations:** 1 KTH MoveAbility, Department of Engineering Mechanics, KTH Royal Institute of Technology, Stockholm, Sweden; 2 Department of Women’s and Children’s Health, Karolinska Institute, Stockholm, Sweden; 3 Faculty of Behavioural and Movement Sciences, VU Amsterdam, Amsterdam, Netherlands

**Keywords:** Ankle exoskeleton, Musculoskeletal simulation, Joint stiffness, Metabolic cost

## Abstract

**Background:**

Ankle-foot exoskeletons can reduce muscle activity and metabolic energy consumption during walking. Experimental studies consistently show that submaximal assistance, typically providing 40–60% of the biological plantarflexion moment, achieves the largest reductions in metabolic power. Inverse musculoskeletal simulations, which neglect factors such as comfort and balance, also predict that submaximal assistance is optimal, yet at higher levels than those observed experimentally. The biomechanical mechanisms underlying this submaximal assistance and its disagreement with experimental observations remain poorly understood.

**Methods:**

In this study, we addressed these questions using inverse simulations of walking and systematically examining muscle-tendon dynamics, metabolic power, and ankle joint stiffness across ten assistance levels (0–100% of the biological plantarflexion moment).

**Results:**

We found that the bi-articular role of the gastrocnemius limits the reduction of metabolic power at the highest levels of assistance. A sensitivity analysis modifying biceps femoris short head strength provided causal confirmation of this mechanism. We further show that increasing assistance substantially reduces ankle joint stiffness by up to 55%, primarily due to de-recruitment of the soleus.

**Conclusions:**

These findings identify both the explanation for submaximal assistance in inverse simulations and propose a missing control objective in exoskeleton simulation frameworks. Because standard effort-based cost functions do not penalize reductions in joint mechanical impedance, this trade-off between effort minimization and stiffness preservation may help explain why simulation-based optimal assistance exceeds experimentally observed values. Inverse simulation does not model robustness, for which impedance preservation may serve as a tractable formulation. Accounting for such a term might represent a new direction for more accurate prediction of optimal exoskeleton assistance.

**Supplementary Information:**

The online version contains supplementary material available at 10.1186/s12984-026-02106-3.

## Background

Ankle-foot exoskeletons have proven highly effective in reducing muscle activations and metabolic power during walking [1, 2]. Experimental studies have identified assistive profiles that reduce the metabolic requirements of walking, typically providing peak moments of ~ 0.6–0.8 Nm/kg, corresponding to approximately 40–50% of the biological ankle plantarflexion moment [1, 3, 4]. Although these optimal profiles vary across individuals and depend on adaptation and training [5], a consistent observation is that submaximal assistance yields the greatest metabolic benefit.

Several explanations can be proposed to explain why submaximal assistance is optimal. These include discomfort due to interface forces [6, 7], disruption of natural motor coordination [8], and changes in muscle mechanics that may reduce force-generating capacity [9, 10]. However, it remains unclear whether these factors alone account for the submaximal optimal or whether other biomechanical constraints intrinsic to the musculoskeletal system play a role. The muscle-level mechanisms that cause metabolic power to rise beyond the optimal assistance have not been characterized, which obscures our understanding of why submaximal, rather than maximal, assistance is beneficial.

Musculoskeletal simulations that neglect subjective and control-related factors consistently predict that submaximal assistance is optimal, suggesting the optimal reflects a structural feature of the musculoskeletal system. Inverse simulation is a widely used approach where joint kinematics and kinetics are imposed, and muscle dynamics are predicted by solving the muscle redundancy problem under effort-based objectives [11, 12], in accordance with the observation that the mechanics of gait is optimal to reduce muscle effort [13, 14]. This approach has been extensively used to examine muscle dynamics with exoskeleton assistance [15–18]. Exoskeleton assistance is typically optimized to minimize muscle activations or metabolic power. The optimal peak assistive moment varies depending on the objective, ranging from ~ 40–50% of the net ankle joint moment for minimizing muscle activations to ~ 70–80% for minimizing metabolic power [15–17, 19]. While inverse simulation predicts that submaximal assistance is optimal, they consistently predict a higher optimal peak moment (1.1–1.2 Nm/kg [17, 19]) than that observed experimentally (0.6–0.8 Nm/kg [3, 5]). Hence, these observations raise two fundamental questions: why do effort-based inverse simulations predict a submaximal optimal assistance level, rather than eliminating muscle activity entirely, and what modeling choice might lead to overestimating its magnitude?

Theoretically, the largest reduction attainable based on effort-based objectives would be to shut down the muscles completely, which is not observed in inverse simulations [17, 19]. At the level of individual muscles, optimal assistance for minimal metabolic power is associated with near-zero soleus activation while the gastrocnemius remains partially active [19]. Despite this coordination being reported, the causal relationship has not been investigated [15, 16]. Soleus and gastrocnemius are the primary contributors to the ankle plantarflexion moment and differ in their anatomy and function [20–22]. Owing to its bi-articular action, the gastrocnemius additionally generates a knee flexion moment during terminal stance [21]. One hypothesis is therefore that partial gastrocnemius activity is required to satisfy the knee flexion demand at terminal stance. The prevalence of gastrocnemius activity would be favored under a minimal-activation recruitment criterion, since its large physiological cross-sectional area makes it well suited to generate combined ankle plantarflexion and knee flexion moments [23, 24]. At high assistance, however, its bi-articular action might generate additional plantarflexion moment that, combined with the assistance, exceeds the required net moment and triggers compensatory antagonist activation, limiting the benefit of offloading the plantarflexors [25]. A second hypothesis is that reducing muscle forces changes muscle-tendon operating conditions, decreasing instantaneous force-generating capacity, characterized by the force-length-velocity relationship [26], and thereby increasing the activation required to produce a given force [9, 10]. These mechanisms were not systematically evaluated across a full range of assistance levels, and their relative contributions to the shape of optimal assistance remain unknown. Identifying which mechanism dominates is a prerequisite for understanding why the predicted optimal is at a higher magnitude than experiments observe.

Furthermore, overestimation of the peak assistive moment suggests that current modeling approaches may omit important constraints. One potential missing factor is the regulation of joint impedance, such as stiffness [27–29], which is known to play a key role in stability and responses to perturbations during walking [30–32]. Standard effort-based cost functions do not explicitly account for joint stiffness and may therefore favor solutions that reduce muscle activity at the cost of substantially decreasing joint mechanical impedance. If the soleus provides a large portion of the ankle joint stiffness, then reducing it might lead to poor regulation of impedance, which is undesirable for dynamic stability. Whether and to what extent this is predicted during exoskeleton assistance remains unknown.

In this study, we used inverse musculoskeletal simulations of walking with an ideal ankle exoskeleton to systematically vary assistance from 0 to 100% of the biological plantarflexion moment and identify why simulations based on an effort-based cost function predict submaximal assistance. First, we tested whether the bi-articular coordination of the gastrocnemius or changes in muscle force-generating capacity explain this optimal. Second, we quantified how assistance changes ankle joint stiffness as an emergent property of muscle dynamics. We hypothesized that (i) persistent gastrocnemius activity due to its bi-articular role, and not reduction in force-generating capacity, explains the submaximal assistance, and (ii) increasing assistance substantially reduces ankle stiffness through reduction of soleus activation, revealing a missing neuromuscular component in current simulation frameworks.

## Methods

### Experimental data, musculoskeletal model, and joint mechanics

We used motion capture and ground reaction force data from a previous study [12]. Briefly, marker trajectories and ground reaction forces were collected from eight participants (6 males, 2 females; age: 39.0 ± 8.6 years; height: 1.77 ± 0.10 m; body mass: 81.1 ± 14.1 kg) walking at 1.29 ± 0.09 m/s at the Promobilia MoveAbility Lab. The study was approved by the Swedish Ethical Review Authority (Dnr. 2020–02311). Marker placement followed the Conventional Gait Model with the extended foot model (CGM 2.4) [33].

Subject-specific musculoskeletal models were generated using OpenSim’s scaling tool [34] based on the model of Delp et al. [23], commonly called the “gait2392” model. The model includes 43 muscles per leg and five degrees of freedom: hip flexion/extension, hip abduction/adduction, hip internal/external rotation, knee flexion/extension, and ankle plantarflexion/dorsiflexion. Joint kinematics and kinetics were computed using OpenSim’s inverse kinematics and inverse dynamics tools. This model and joint kinematics and kinetics are subsequently used as inputs in inverse simulations.

### Inverse simulations

We used an inverse simulation approach to study the effect of an ideal ankle plantarflexion exoskeleton on simulated muscle dynamics [35]. The inverse method used joint kinematics and kinetics derived from experimental data of subjects walking without an exoskeleton, under the assumption that the addition of an exoskeleton does not alter these patterns. By fixing joint mechanics, the analysis isolates muscle dynamics from gait adaptations that may occur with assistance.

Muscle control, states, and state derivatives are computed by solving an optimization problem. The cost function to solve the muscle redundancy problem minimizes the sum of squared muscle activations, a common criterion for simulating gait [11, 12]. The exoskeleton assistance was modeled as an ideal moment actuator $\:{\tau\:}_{E}$ applied to the ankle joint. At each time step, the sum of muscle moments $\:{\tau\:}_{{M}_{j}}$ and exoskeleton moment $\:{\tau\:}_{E}$ was constrained to match the inverse dynamics joint moments $\:{\tau\:}_{I{D}_{j}}$:

1 $$\:{\tau}_{I{D}_{j}}\left(t\right)={\tau}_{{M}_{j}}\left(t\right)+{\tau}_{E}\left(t\right)$$

2 $$\:{\tau}_{{M}_{j}}\left(t\right)=\sum\:{r}_{ij}\left(t\right){F}_{{M}_{i}}\left(t\right)$$

Where $\:{r}_{ij}$ is the moment arm of the muscle $\:i$ at the joint $\:j$, and $\:{F}_{{M}_{i}}$ is the muscle force of the muscle *i*. We simulated walking without assistance (i.e., $\:{\tau}_{E}=0$) and with nine assistive moments ranging from 10% to 100%, in increments of 10%, of the net ankle plantarflexion moment computed from inverse dynamics (i.e., $\:{\tau}_{E}$ is a percentage of $\:{\tau}_{ID}$). The trajectory of the assistive moment over the gait cycle therefore reproduced the shape of the net ankle plantarflexor moment profile at scaled magnitudes, where the total joint moment, generated by muscles plus exoskeleton, equaled the inverse dynamics solution derived from unassisted walking at every time step.

### Simulation metrics: muscle-tendon dynamics, metabolic power, and joint stiffness

Muscle-tendon dynamics, including muscle activations, tendon forces, fiber lengths, and fiber velocities, were obtained from the solution of the muscle redundancy problem.

The net metabolic power $\:{E}_{NET}$ is based on the estimated muscle metabolic power $\:{E}_{M}$. We used an energy model described by Bhargava et al. [36], which combines muscle mechanical power and heat dissipation rate. The mechanical power was computed as the product of muscle force and fiber velocity. The heat dissipation rate was modeled as a function of muscle mass, activation, and fiber velocity. We also adjusted the heat dissipation rate to ensure non-negative metabolic power [37]. Net metabolic power was the sum of the metabolic power of all muscles:

3 $$\:{E}_{NET}\left(t\right)=\sum\:_{i=1}^{{N}_{M}}{E}_{{M}_{i}}\left(t\right)dt$$

Joint stiffness $\:K$ was calculated by deriving its time-varying mechanical impedance using musculoskeletal dynamics [32]. The muscle stiffness $\:{K}_{{M}_{i}}^{j}$ at the joint $\:j$ for each the muscle$\:\:i$ was derived from its muscle-tendon stiffness $\:{K}_{MTU}$ and muscle forces $\:{F}_{T}$:

4 $$\:{K}_{{MTU}_{i}}=\left(\frac{1}{{K}_{{T}_{i}}}+\frac{1}{{K}_{{M}_{i}}^{EFF}}\right)$$

5 $$\:{K}_{{M}_{i}}^{EFF}={K}_{{M}_{i}}co{s}^{2}{\alpha\:}_{i}+\frac{{F}_{{M}_{i}}si{n}^{2}{\alpha\:}_{i}}{{l}_{{M}_{i}}}$$

6 $$\:{K}_{{M}_{i}}^{j}\left(t\right)={K}_{{MTU}_{i}}\left(t\right){r}_{ij}^{2}\left(t\right)+\frac{\partial\:{r}_{ij}}{\partial\:{\theta\:}_{j}}\left(t\right){F}_{{T}_{i}}\left(t\right)$$

7 $$\:{K}^{j}\left(t\right)=\sum\:_{i=1}^{{N}_{M}}{K}_{{M}_{i}}^{j}\left(t\right)$$

Where $\:{K}_{{T}_{i}}$ is tendon stiffness, $\:{K}_{{M}_{i}}$ is the muscle stiffness, $\:{K}_{{M}_{i}}^{EFF}$ is the effective muscle stiffness, and $\:\alpha\:$ is the pennation angle at the muscle $\:i$, and $\:{\theta\:}_{j}$ is the angle at the joint $\:j$. The tendon stiffness $\:{K}_{T}$ was computed as the derivative of the tendon force-strain relationship. The muscle stiffness $\:{K}_{M}$ was computed numerically as the partial derivative of muscle force with respect to fiber length, incorporating both active (i.e., activation and force-length-velocity multiplier) and passive contributions. The effective muscle stiffness $\:{K}_{M}^{EFF}$ is the projection onto the tendon axis, accounting for pennation angle $\:\alpha\:$ .

### Data analysis

For each level of assistance, we computed mean muscle activations, metabolic power, muscle-tendon dynamics, and ankle joint stiffness. Mean values $\:{X}_{avg}$ were obtained by integrating the variable of interest over the gait cycle (or stance phase for stiffness) and divided by the duration of the interval $\:T$:

8 $$\:{X}_{avg}=\frac{1}{T}{\int\:}_{{t}_{i}}^{{t}_{f}}X\left(t\right)dt$$

Where $\:{t}_{i}$ and $\:{t}_{f}$ is the initial and final period of analysis, respectively.

We evaluated the relationship between mean muscle activation $\:{a}_{avg}$, calculated by including all muscles $\:a$ in a leg, and metabolic power $\:{E}_{avg}$, calculated using the time series metabolic rate $\:{E}_{NET}$, across levels of assistance, and identified the level of assistance that yielded the optimal reduction. The relationship between predicted outcomes and levels of assistance was characterized by using a third-order polynomial fit. The optimal level of assistance was defined as the minimum of the fitted curve. Visual inspection confirmed that the polynomial model adequately captured the observed non-linear trends.

To investigate the mechanisms responsible for suboptimal assistance, we evaluated the mean values of muscle activation, tendon force, metabolic power, normalized fiber length, and fiber velocity over the gait cycle across all subjects. We also evaluated the instantaneous force muscle capacity, defined as the force-length-velocity relationship [26]. To further isolate the contribution of bi-articular gastrocnemius coordination, we conducted a sensitivity analysis modifying the maximum isometric force of the biceps femoris short head, the only uniarticular knee flexor in the model. By systematically scaling this muscle’s strength by +/- 50% of its value, we altered the model’s capacity to satisfy the knee flexion moment without gastrocnemius contribution, thereby testing whether modifying the gastrocnemius during knee flexion shifts the predicted optimal assistance.

To investigate the effect of joint stiffness across levels of assistance, we evaluated the mean joint stiffness over the stance phase across all subjects. Furthermore, we computed the contribution of stiffness from the major muscles around the ankle joint and evaluated their changes across the level of assistance.

A large language model (Claude, Anthropic, San Francisco, CA, USA) was used for grammar and language editing of the manuscript. All scientific content, analyses, and interpretations are exclusively from the authors.

## Results

**Fig. 1:**
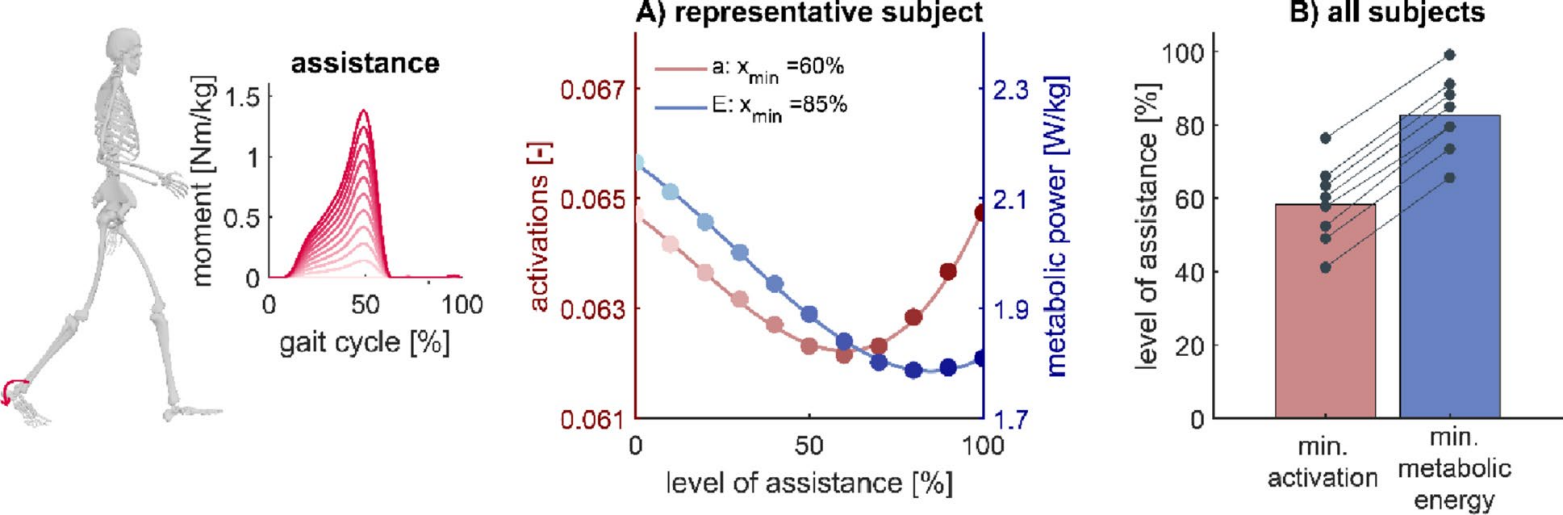
Submaximal assistance resulted in the largest reductions in average muscle activity and metabolic power. Values across levels of assistance in a representative subject (**A**) and optimal levels of assistance in all subjects (**B**). Each dot represents an individual subject in plot B

**Fig. 2:**
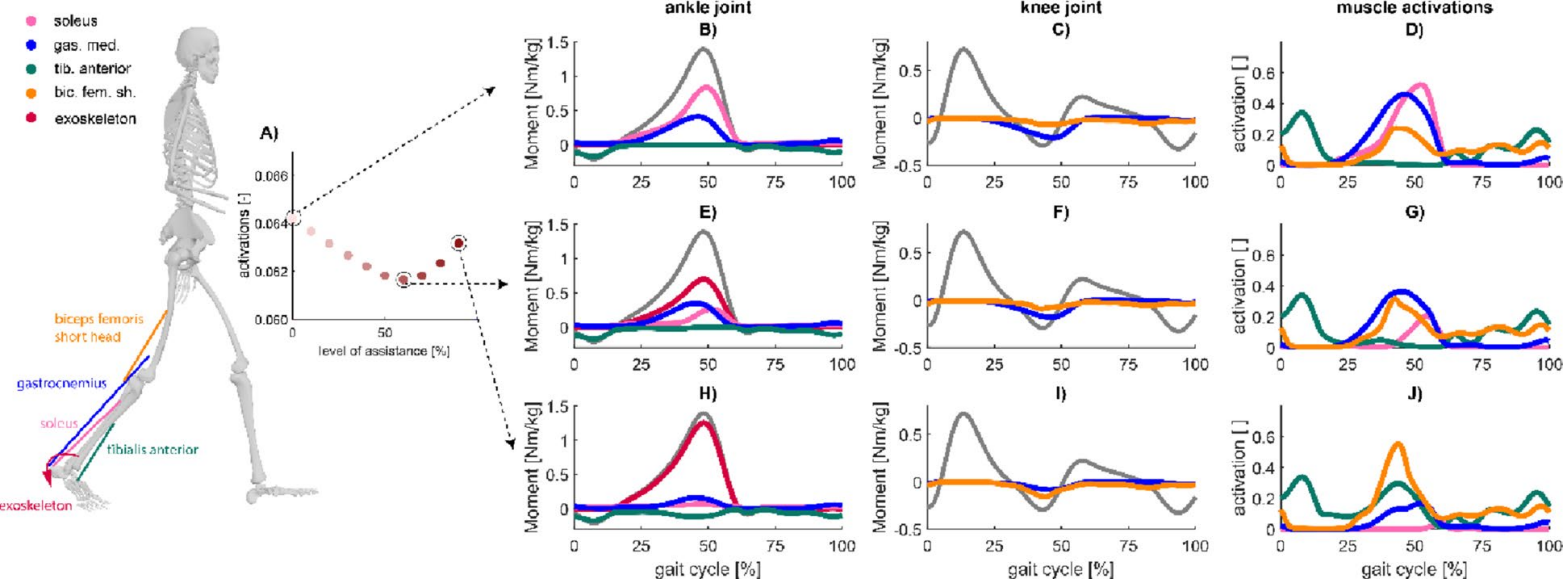
Changes in muscle moments and activations. Submaximal assistance resulted in the largest reductions in average muscle activations, as shown in a representative subject (**A**). Joint and muscle moments at the ankle (**B**,** E**, and** H**) and knee joints (**C**,** F**, and** I**) and muscle activations (**D**,** G**, and** J**) during walking with 0, 60, and 90% of the maximum level of assistance for one representative subject. Muscle moments of soleus (pink line), gastrocnemius medialis (blue line), tibialis anterior (green line), and biceps femoris short head (orange line) spanning the ankle and knee joint, and exoskeleton moment (red line) were shown

Submaximal ankle assistance minimized muscle activations at ~ 60% and metabolic power at ~ 80% of the net plantarflexion moment (Fig. 1). This observation was consistent across all subjects (Supplementary Fig. 1) and was primarily associated with the bi-articular function of the gastrocnemius (Fig. 2). In unassisted conditions, gastrocnemius contributed to both ankle plantarflexion and knee flexion moments during mid-stance and pre-swing. As assistance increased, the required ankle plantarflexion moment generated by muscles decreased while the knee flexion moment remained invariant. Consequently, soleus moment approached zero and gastrocnemius moment decreased, although some gastrocnemius activation was required to satisfy the knee flexion moment at every level of assistance (Fig. 2C, F, I). As assistance increased, the combined plantarflexion moment of the exoskeleton and gastrocnemius exceeded the required net ankle moment, necessitating compensatory tibialis anterior activation to maintain the net ankle moment (Fig. 2J). Excessive exoskeleton assistance also increased activations in knee flexors, such as the biceps femoris short head, compensating for the decreased contribution of the gastrocnemius to knee flexion (Fig. 2H).

**Fig. 3:**
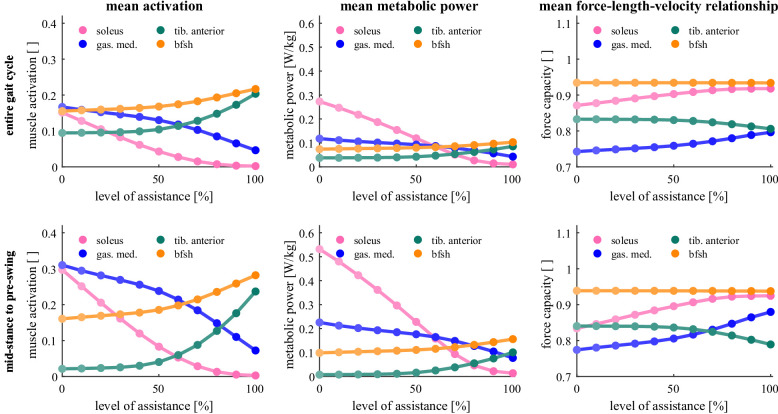
Mean muscle activations, metabolic rates, and force-length-velocity relationship of soleus, gastrocnemius medialis, tibialis anterior, and biceps femoris short head (bfsh) muscles across levels of assistance. Mean values were computed with respect to the entire gait cycle (upper row) and mid-stance to pre-swing phase, 12 to 62% of the gait cycle. The force-length-velocity relationship represents the active force generation capacity of the muscle, computed as the product of the force-length and force-velocity relationship. Average values were computed among all subjects

The difference in optimal assistance level between effort-based objectives was a byproduct of the compensatory strategies and how each objective weights muscle contributions. At optimal assistance for minimal activations, soleus was nearly suppressed and gastrocnemius reduced by ~ 20% compared to unassisted conditions; further increases in assistance decreased gastrocnemius at the expense of increasing tibialis anterior and biceps femoris short head, which raised the overall activations (Fig. 3). At optimal assistance for minimal metabolic power, a higher assistance level was sustained because the benefit of further fully suppressing soleus and decreasing gastrocnemius outweighed the metabolic power of recruiting compensatory muscles (Fig. 3).

**Fig. 4:**
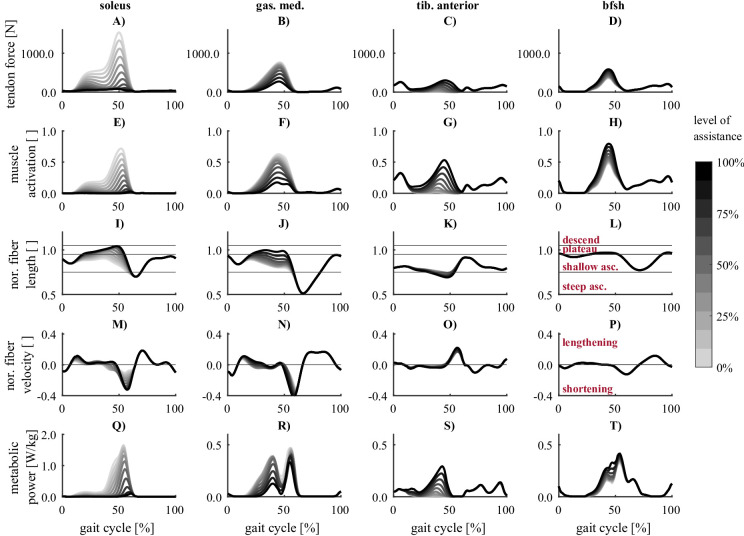
Exoskeleton assistance influences muscle activations, states, and metabolic power. Tendon force, muscle activation, normalized fiber length, normalized fiber velocity, and metabolic power of soleus, gastrocnemius medialis (gas. med.), tibialis anterior (tib. anterior), and biceps femoris short head (bfsh) across levels of assistance. Average values computed among all subjects

Exoskeleton assistance influenced operating lengths and velocities, but changes in force-generating capacity did not explain the submaximal assistance. In unassisted conditions, gastrocnemius and soleus generated moments during stance while operating at the shallow ascent of the force-length relationship and with relatively small fiber length excursions (Fig. 4I, J, M, and N). As assistance increased, the tendon force and, therefore, tendon length decreased. As the muscle-tendon length, derived from joint kinematics, was imposed in the inverse simulations, this resulted in increased muscle fiber length changes and, hence, velocities (especially at push-off), which were associated with lower instantaneous force capacity (Fig. 3). However, the soleus and gastrocnemius also operated closer to the optimal length during midstance with increased assistance, which was associated with higher instantaneous force capacity. As a result, the force-length-velocity multiplier in plantarflexors slightly increased with higher exoskeleton assistance (Figs. 3 and 4); hence, changes in force-generating capacity were not the primary mechanism why the submaximal assistance was optimal. Conversely, tibialis anterior instantaneous force capacity declined with assistance: the muscle generated larger forces that drove fibers toward the steep ascending limb (Fig. 4G, K, O). Biceps femoris short head force capacity remained largely unchanged relative to unassisted walking (Figs. 3 and 4H, L, P).

**Fig. 5:**
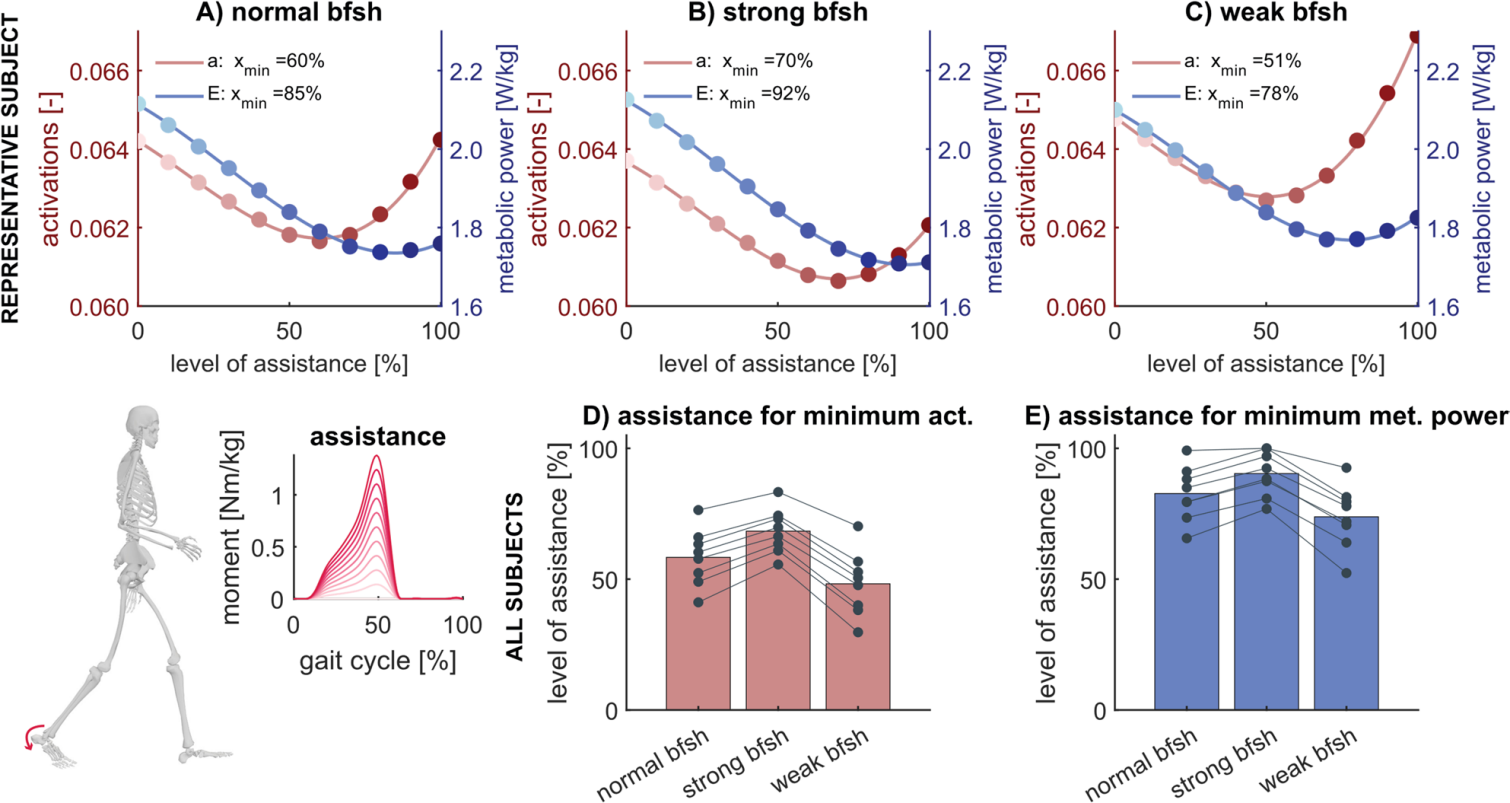
Biceps femoris strength influenced the optimal level of assistance to reduce muscle activations and metabolic power. Increased biceps femoris strength (150% $\:{F}_{M}^{0}$) increased the optimal level of assistance and reduced biceps femoris strength (weak: 50% $\:{F}_{M}^{0})$ had the opposite effect, as shown in a representative subject (**A**,** B**, and** C**). Mean muscle activations $\:{a}_{avg}$ (**D**) and metabolic power $\:{\dot{E}}_{avg}$ (**D**) were computed across all the muscles in the musculoskeletal model. Each dot represents an individual subject in the plots **D** and **E**

Modifying biceps femoris short head strength changed the relative contribution of gastrocnemius to generating knee flexion moment and confirmed the mechanistic role of bi-articular gastrocnemius coordination. When the biceps femoris short head strength was increased, the gastrocnemius’s contribution to knee flexion decreased, allowing for higher exoskeleton assistance without tibialis anterior compensation; hence, the optimal assistance was higher in this condition compared to the biceps femoris short head at nominal strength. Conversely, weaker biceps femoris short head strength increased the gastrocnemius’s role in knee flexion, limiting the benefits of higher exoskeleton assistance (Fig. 5).

**Fig. 6:**
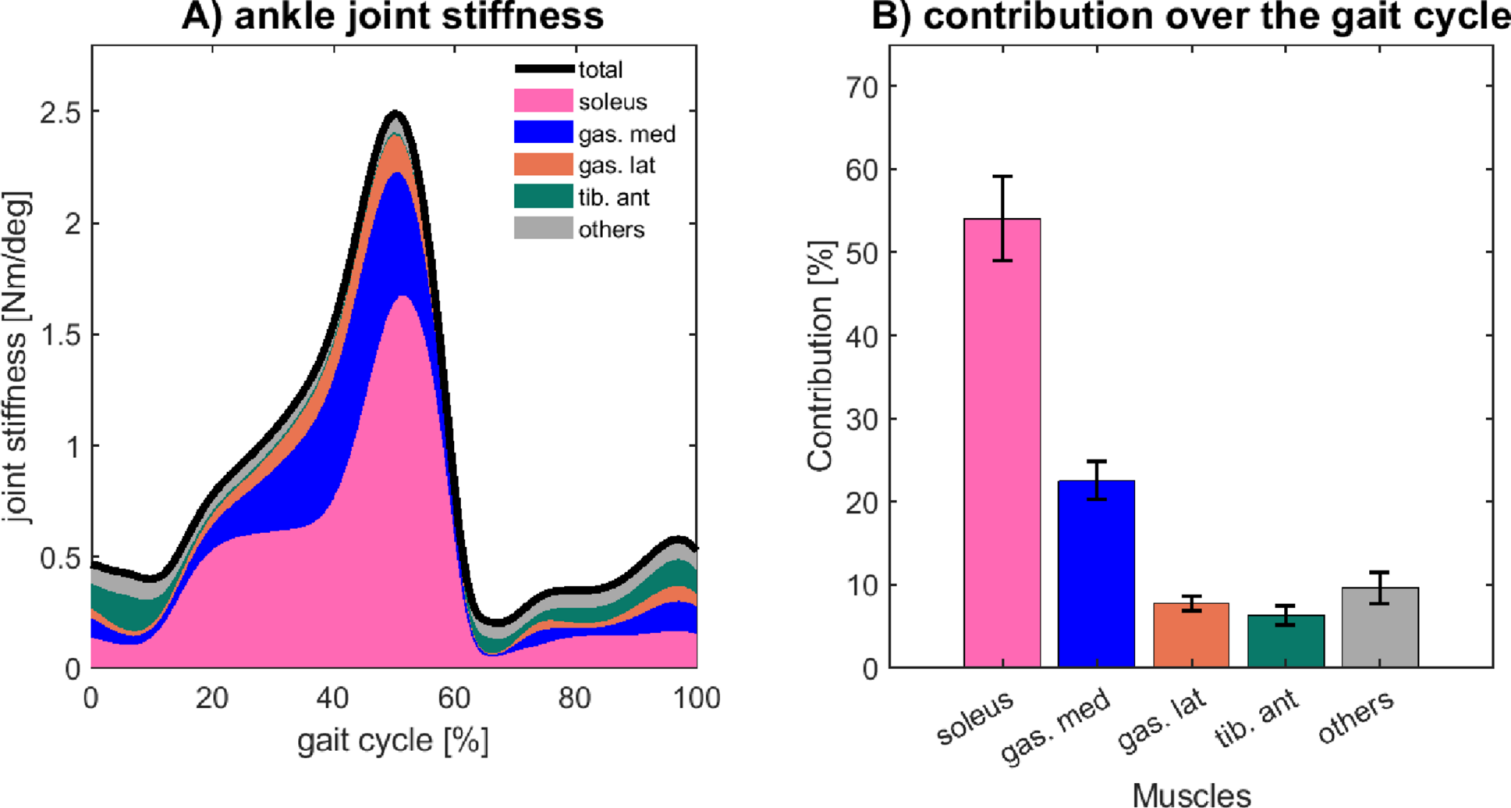
Ankle joint stiffness and contribution of major muscles in unassisted conditions. Time series of the ankle joint stiffness and the major muscles around the ankle (**A**) and the percentage contribution of individual muscles around the ankle (**B**). Average values computed among all subjects. Vertical bar at each muscle represents one standard deviation in plot **B**

Ankle joint stiffness exhibited a characteristic time-varying profile across the gait cycle. Stiffness increased progressively after loading response and reached a peak at 50% of the gait cycle (Fig. 6A). The relative contributions of individual muscles revealed that the soleus was the dominant contributor to ankle joint stiffness under unassisted conditions, accounting for ~ 55% of the total stiffness (Fig. 6B). The gastrocnemius medialis contributed by ~ 20%, while smaller contributions were observed from the gastrocnemius lateralis and tibialis anterior.

**Fig. 7:**
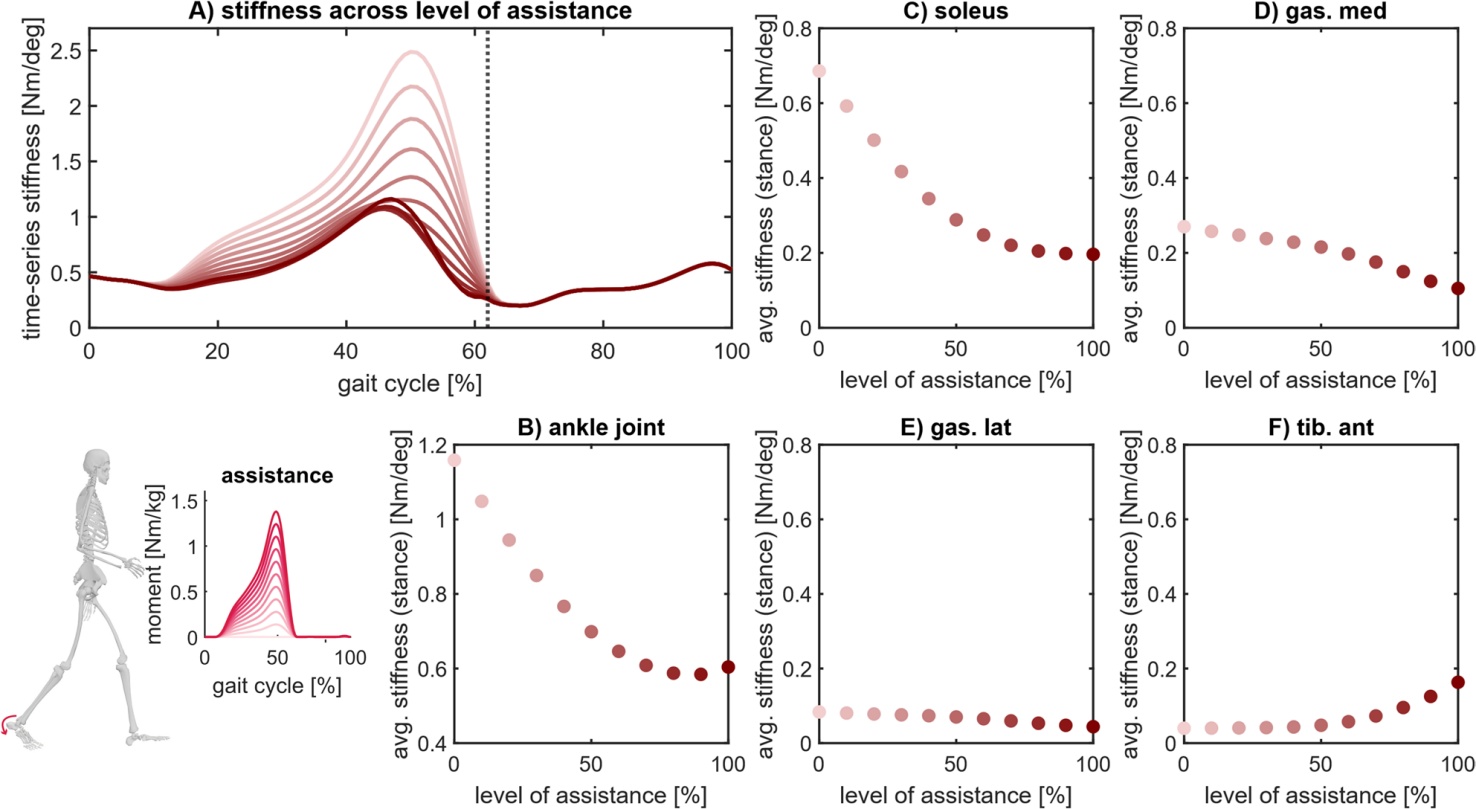
Ankle and muscle joint stiffness across the level of assistance. Time series ankle joint stiffness across levels of assistance (**A**), and mean values, over the stance phase, of the ankle joint stiffness (**B**), soleus (**C**), gastrocnemius medialis (**D**), gastrocnemius lateralis (**E**), and tibialis anterior (**F**). Average values computed among all subjects

Increasing exoskeleton assistance substantially reduced the ankle joint stiffness during the stance phase. The peak stiffness decreased progressively with increasing exoskeleton assistance, leading to up to ~ 55% reduction compared to unassisted conditions (Fig. 7A). Within the stance phase, the ankle joint stiffness decreased by approximately 50% at high exoskeleton assistance, i.e., ~ 90% of the net plantarflexion moment (Fig. 7B), associated primarily with the reduction of the soleus stiffness (Fig. 7C). Its value decreased with increasing exoskeleton assistance, dropping by 65%, compared to unassisted conditions, at ~ 70% of the net plantarflexion moment, after which it plateaued. At those levels of assistance, soleus continued to provide passive forces as its fiber length is higher than the optimal length (Fig. 4I). Such a small force still contributed to the ankle stiffness, even when the muscle is shut down. The gastrocnemius medialis and lateralis also showed a reduction in stiffness, although to a lesser extent and more gradually as the assistance increases (Fig. 7D and E). In contrast, the tibialis anterior stiffness increased at moderate exoskeleton assistance, reflecting its compensatory activation (Fig. 7F); however, this increase only slightly offset the substantial reduction led by plantarflexors.

## Discussion

The bi-articular function of the gastrocnemius explains why partial, rather than complete, ankle plantarflexion moment assistance was optimal for reducing muscle activations and metabolic power in inverse walking simulations. As exoskeleton assistance increased, soleus activation approached zero, yet the gastrocnemius remained partially active to satisfy the invariant knee flexion moment. Beyond the optimal assistance level, compensatory activations of tibialis anterior and biceps femoris short head increased both muscle activations and metabolic power. Instantaneous force-generating capacity in plantarflexors slightly increased with assistance and therefore did not constitute the primary mechanism to explain why submaximal assistance was optimal. Soleus was the dominant contributor to ankle joint stiffness, and its decreased activation, with increasing assistance, produced a near-monotonic reduction in joint mechanical impedance, an emergent consequence of effort-based cost function minimization.

The bi-articular role of the gastrocnemius as a limiting factor in maximal plantarflexion assistance is partially consistent with experimental observations. Consistent with experimental findings [38–40], optimal plantarflexion assistance primarily decreased soleus activity and, to a lesser extent, gastrocnemius activity. In a human-in-the-loop optimization study minimizing metabolic power [40], peak soleus activation was reduced by 45% and peak gastrocnemius activation by 30%, indicating that the gastrocnemius remained active during assisted gait. Following this reasoning, simultaneously assisting the ankle and knee joint should reduce gastrocnemius activation further by decreasing its knee flexion contribution. However, optimized ankle-knee exoskeleton assistance did not result in larger reductions in gastrocnemius activation compared to ankle-only assistance, nor in larger optimal plantarflexion moments [41]. This observation indicates that some gastrocnemius activity is preserved during experimental assisted gait, but the mechanism differs from the bi-articular coordination predicted by inverse simulations. Experimentally, this preservation did not involve compensatory increases in tibialis anterior or biceps femoris short head activation [4, 40]. The inverse simulation, therefore, captures the qualitative constraint correctly: partial gastrocnemius activity persists, but through a different causal pathway than the one the nervous system employs. No study has characterized muscle dynamics and joint mechanics across assistive loads that exceed the experimentally observed optimal; exploring the neuromechanical adaptation by means of empirical observations [40] or simulation-informed approaches [9] would provide a foundational understanding of the optimal human-robot interaction.

The modeled trajectory of the assistive moment or choice of metabolic cost model is unlikely to explain the disagreement with human-in-the-loop experiments. In a previous simulation study [19], we optimized assistance for minimal metabolic power based on a four-parameter moment-time profile as Zhang et al. [3] commonly used in human-in-the-loop experiments [1, 3, 4]. We found the same qualitative findings, where at the optimal assistance, soleus activation was suppressed, and the gastrocnemius activation decreased. Both the scaled ankle plantarflexion profile, as used in this study, and the moment-time profile enabled complete reduction of soleus, predicting optimal assistance that exceeded experimentally observed values. Soleus is the primary driver of metabolic power during walking within typically used metabolic energy models [42]; its suppression to minimize metabolic power is therefore expected regardless of the metabolic modeling assumptions. Compensatory strategies, i.e., an increase of tibialis anterior or biceps femoris short head, limit the optimal assistive magnitude, yet they only emerge once soleus is substantially suppressed, a coordination pattern not observed experimentally. Together, these findings indicate that the disagreement with human-in-the-loop experiments reflects a structural property of effort-based cost functions rather than modeling assumptions about torque trajectory or metabolic energy.

The predicted ankle joint stiffness profile at unassisted conditions is consistent with experimental measurements. Rouse et al. reported an increase in ankle stiffness between mid- and terminal stance consistent with our predictions; specifically, our estimated stiffness of 0.019 Nm/deg/kg at 40% of the gait cycle falls within their reported range of 0.025 ± 0.007 Nm/deg/kg at a comparable walking speed [31]. Lee and Hogan reported that stiffness decreased substantially before toe-off, remained relatively constant during swing, and increased before heel strike [30], a trajectory consistent with our time-varying profiles, though reported at a lower walking speed of ~ 0.8 m/s. Sartori et al. reported a similar stiffness trajectory during stance at a higher speed (~ 1.9 m/s) and identified the soleus as the primary contributor to ankle joint stiffness [32], consistent with our finding. Across these comparisons, effort-based inverse simulations reproduced the known features of ankle joint stiffness during unassisted walking. Exact quantitative agreement was not expected, as other motor control strategies such as antagonist co-contraction, which increase joint impedance beyond what effort minimization predicts [43], were not modeled; additionally, the Hill-type formulation captures instantaneous mechanical stiffness but not time-history-dependent contributions, which may further influence stiffness estimates during cyclic contractions [44]. The reported values should therefore be interpreted as a lower bound, suitable for characterizing qualitative changes across assistance levels. No experimental data have reported how ankle joint stiffness changes across levels of exoskeleton assistance. Future studies might consider characterizing such a relationship through experiments or neuromusculoskeletal approaches.

The substantial reduction in ankle joint stiffness with increasing exoskeleton assistance is an emergent mechanical consequence of effort minimization. Because the soleus is simultaneously the primary metabolic contributor during push-off and the dominant source of ankle joint stiffness, suppressing it to minimize effort inevitably compromises joint mechanical impedance, a trade-off the neuromuscular system is unlikely to accept freely [45]. The experimental observation that soleus activation was only partially reduced, even in fully trained exoskeleton users [4, 40], was consistent with the nervous system preserving ankle impedance above a functional threshold. This threshold cannot be determined by muscle effort minimization alone and must instead reflect additional motor control objectives related to stability and perturbation rejection [46]. This structural omission in standard inverse simulation formulations is therefore a plausible explanation for why predicted optimal assistance magnitude consistently overestimates the experimentally observed value: the optimizer silenced the soleus beyond what the neuromuscular system would accept before compromising joint stability. Future studies should test this hypothesis directly by measuring ankle joint stiffness via musculoskeletal dynamics [46] or perturbation responses [31] across a range of exoskeleton assistance levels in both trained and untrained users.

These findings motivate extending inverse simulation cost functions to account for joint impedance regulation. Ankle impedance may emerge from complex interactions between sensorimotor noise, environmental uncertainty, feedback control, and stability requirements during walking, which can be captured under stochastic optimal control simulation [47]. However, this approach remains computationally intensive, which currently limits its feasibility for large-scale exoskeleton design optimization or rapid assistance parameter exploration. As a more tractable alternative, multi-objective formulations that minimize both muscle effort and deviations from a baseline stiffness profile may predict lower optimal assistance levels that better align with experimentally observed values. Alternatively, a minimum acceptable ankle impedance could be enforced as a constraint at each phase of stance, requiring normative stiffness data as a reference, for which the perturbation-based impedance literature provides a foundation [30, 31]. Muscle synergy-based control offers a complementary path, as synergies naturally promote coactivation patterns that increase joint stiffness [43, 48]. These strategies might help to better predict optimal control, complementing musculoskeletal simulation approaches based on minimal effort.

In this study, we deliberately employed inverse simulation to examine the mechanical principles underlying submaximal assistance and to quantify how ankle joint stiffness changes across levels of exoskeleton assistance. By holding kinematics fixed, this approach isolated the behavior of the effort-minimizing cost function from gait adaptations that accompany real exoskeleton use, which was needed to identify what effort minimization can and cannot account for. A consequent limitation was that motion adaptation was not permitted. Joint kinematics and kinetics are expected to vary with exoskeleton assistance, with the magnitude of adaptation differing across joints and depending on the torque trajectory [49, 50]. Accounting for these changes would provide a more complete description of human-robot adaptation, but introduces confounding factors. In predictive simulations, kinematics and kinetics emerge from the interplay of multiple terms in the objective function [51]. Prior work has demonstrated that predicted gait mechanics are sensitive to the choice of objective function [52, 53], and incorporating assistive moments results in motion mechanics that differ substantially from unassisted conditions, which is not observed experimentally [54–56]. Analysis with inverse simulations implies negligible changes in joint mechanics, an assumption that, as in this study, helps to identify structural limitations of effort-minimizing cost functions. Identifying the governing principles of human-robot adaptation remains an open challenge and a critical direction for future research [57].

Whether the stiffness-effort trade-off identified here persists when gait mechanics adapt to assistance remains an open question, but existing evidence points in that direction. Nguyen et al. predicted optimal ankle exoskeleton assistance using a predictive simulation with a multi-objective cost function that minimizes muscle fatigue and enforces a periodic gait solution (limit cycle) by penalizing displacement in the center of mass, head position, and base of support [55]. Even with this formulation that involved gait stability metrics, the predicted peak assistive moment reached 1.5 Nm/kg, exceeding experimentally reported optimal [1, 3, 4] by a similar margin as in inverse simulations, which only minimize muscle effort. This finding suggests that overestimation of optimal assistance is not an artifact of fixed kinematics or single-objective effort minimization alone but may instead reflect a broader gap in how optimality is formulated. With predictive simulations, muscle coordination might follow a recruitment pattern that results in more accurate prediction of some gait mechanics, e.g., higher ankle plantarflexion push-off compared to the unassisted condition [55, 56], and yet yield near-zero muscle activations if the objective function favors minimal effort solutions without accounting for local dynamic stability [47].

What is ultimately missing is a term that captures gait robustness: the capacity to maintain a periodic solution under perturbation, which effort minimization alone does not encode. Incorporating robustness explicitly, for example, through stochastic or robust optimal control, remains computationally demanding; constraining mechanical impedance, and joint stiffness in particular, may serve as a tractable proxy, since preserving impedance under assistance is likely to condition the system against perturbation-induced deviations. Future predictive simulations would benefit from investigating how joint impedance evolves with assistance level, and from testing whether incorporating impedance constraints or robustness terms yields more realistic optimal assistance profiles. Incorporating reflex-mediated contributions represents a further extension to capture neuromuscular impedance regulation beyond what voluntary activation and passive mechanics alone provide [46]. In the context of assisted gait, however, this requires first characterizing how sensory information changes relative to unassisted conditions, since afferent signals, which modulate reflex gain and joint stiffness [45], may differ substantially between unassisted and assisted walking [58], posing the challenge of identifying whether and how the neuromuscular system adapts its feedback control strategy to the altered mechanical context. Establishing the relationship between neuromuscular commands and muscle forces would represent a substantive step toward closing the gap between computational predictions and experimental observations.

## Conclusions

This study explains why effort-based musculoskeletal simulations predict submaximal ankle exoskeleton assistance and why they might systematically overestimate peak assistive moments. We show that the submaximal assistance emerges primarily from bi-articular coordination: gastrocnemius remains active to satisfy knee flexion demands, and excessive assistance requires compensatory forces from ankle dorsiflexion and knee flexion. We further showed that increasing assistance substantially reduces ankle joint stiffness by up to ~ 55%, primarily through the reduction of soleus activation, a consequence that current effort-based cost functions do not penalize. These findings suggest that the mismatch between simulated and experimental observations may not stem solely from adaptation or comfort limitations, but from missing neuromechanical objectives in current models. Incorporating impedance regulation into future simulation frameworks may improve the in silico design of wearable robots and better align computer simulations with human motor behavior.

## Supplementary Information

Below is the link to the electronic supplementary material.

Supplementary Material 1

## Data Availability

Processed experimental data: Scaled musculoskeletal models, joint kinematics, and joint kinetics, as well as the simulation framework, are available in a public repository: https://github.com/israelluis/GoalOptimized.

## Abbreviations

CGM: Conventional gait model
MTU: Muscle-tendon unit
*K*: Joint stiffness
*K*_*MTU*_: Muscle-tendon unit stiffness
*K*_*M*_: Muscle stiffness
${K}_{MTU}^{\mathrm{E}\mathrm{F}\mathrm{F}}$: Effective muscle stiffness
*K*_*T*_: Tendon stiffness
*F*_*M*_: Muscle force
*F*_*T*_: Tendon force
${r}_{ij}$: Moment arm of muscle i at joint j
$\:{\tau}_{E}$: Exoskeleton assistive moment
${\tau}_{M}$: Net muscle moment
${\tau}_{ID}$: Inverse dynamics joint moment
${E}_{NET}$: Net metabolic power
${E}_{M}$: Muscle metabolic power
$$\alpha\:$$: Pennation angle
$$\:\theta\:$$: Joint angle
BFSH: Biceps femoris short head
SOL: Soleus
GAS: Gastrocnemius
TA: Tibialis anterior

## References

[1] Slade P, Kochenderfer MJ, Delp SL, Collins SH. Personalizing exoskeleton assistance while walking in the real world. Nature. 2022;610(7931):7931. 10.1038/s41586-022-05191-1. 10.1038/s41586-022-05191-1 PMC9556303 36224415

[2] Sawicki GS, Beck ON, Kang I, Young AJ. The exoskeleton expansion: improving walking and running economy. J Neuroeng Rehabil. 2020;17(1):25. 10.1186/s12984-020-00663-9. 32075669 10.1186/s12984-020-00663-9 PMC7029455

[3] Zhang J, Fiers P, Witte KA, Jackson RW, Poggensee KL, Atkeson CG, et al. Human-in-the-loop optimization of exoskeleton assistance during walking. Science. 2017;356(6344):1280–3. 10.1126/science.aal5054. 28642437 10.1126/science.aal5054

[4] Franks PW, Bryan GM, Martin RM, Reyes R, Lakmazaheri AC, Collins SH. Comparing optimized exoskeleton assistance of the hip, knee, and ankle in single and multi-joint configurations. Wearable Technol. 2021;2. 10.1017/wtc.2021.14. 10.1017/wtc.2021.14 PMC10936256 38486633

[5] Poggensee KL, Collins SH. How adaptation, training, and customization contribute to benefits from exoskeleton assistance. Sci Robot. 2021;6:1078. 10.1126/scirobotics.abf1078. 10.1126/scirobotics.abf1078 34586837

[6] Chen L, Zhou D, Leng Y. A Systematic Review on Rigid Exoskeleton Robot Design for Wearing Comfort: Joint Self-Alignment, Attachment Interface, and Structure Customization. IEEE Trans Neural Syst Rehabil Eng. 2024;32:3815–27. 10.1109/TNSRE.2024.3479283. 39401109 10.1109/TNSRE.2024.3479283

[7] Yandell MB, Ziemnicki DM, McDonald KA, Zelik KE. Characterizing the comfort limits of forces applied to the shoulders, thigh and shank to inform exosuit design. PLoS ONE. 2020;15(2):e0228536. 10.1371/journal.pone.0228536. 32049971 10.1371/journal.pone.0228536 PMC7015417

[8] Steele KM, Jackson RW, Shuman BR, Collins SH. Muscle recruitment and coordination with an ankle exoskeleton. J Biomech. 2017;59:50–8. 10.1016/j.jbiomech.2017.05. .010 PubMed PMID: 28623037. 28623037 10.1016/j.jbiomech.2017.05.010 PMC5644499

[9] Jackson RW, Dembia CL, Delp SL, Collins SH. Muscle–tendon mechanics explain unexpected effects of exoskeleton assistance on metabolic rate during walking. J Exp Biol. 2017;220(11):2082–95. 10.1242/jeb.150011. 28341663 10.1242/jeb.150011 PMC6514464

[10] Nuckols RW, Dick TJM, Beck ON, Sawicki GS. Ultrasound imaging links soleus muscle neuromechanics and energetics during human walking with elastic ankle exoskeletons. Sci Rep. 2020;10(1):3604. 10.1038/s41598-020-60360-4. 32109239 10.1038/s41598-020-60360-4 PMC7046782

[11] Zargham A, Afschrift M, De Schutter J, Jonkers I, De Groote F. Inverse dynamic estimates of muscle recruitment and joint contact forces are more realistic when minimizing muscle activity rather than metabolic energy or contact forces. Gait Posture. 2019;74:223–30. .019 PubMed PMID: 31563823. 31563823 10.1016/j.gaitpost.2019.08.019

[12] Luis I, Afschrift M, De Groote F, Gutierrez-Farewik EM. Evaluation of musculoskeletal models, scaling methods, and performance criteria for estimating muscle excitations and fiber lengths across walking speeds. Front Bioeng Biotechnol. 2022;10. 10.3389/fbioe.2022.1002731. 10.3389/fbioe.2022.1002731 PMC9583830 36277379

[13] Crowninshield RD, Brand RA. A physiologically based criterion of muscle force prediction in locomotion. J Biomech. 1981;14(11):793–801. 10.1016/0021-9290(81)90035-X. 7334039 10.1016/0021-9290(81)90035-x

[14] McNeill Alexander R. Energetics and optimization of human walking and running: the 2000 Raymond Pearl memorial lecture. Am J Hum Biol. 2002;14(5):641–8. 10.1002/ajhb.10067 . PubMed PMID: 12203818. 12203818 10.1002/ajhb.10067

[15] Dembia CL, Silder A, Uchida TK, Hicks JL, Delp SL. Simulating ideal assistive devices to reduce the metabolic cost of walking with heavy loads. PLoS ONE. 2017;12(7):e0180320. 10.1371/journal.pone.0180320. 28700630 10.1371/journal.pone.0180320 PMC5507502

[16] Luis I, Afschrift M, Gutierrez-Farewik EM. Springs vs. motors: Ideal assistance in the lower limbs during walking at different speeds. PLoS Comput Biol. 2024;20(9):e1011837. 10.1371/journal.pcbi.1011837. 39231195 10.1371/journal.pcbi.1011837 PMC11404844

[17] Bianco NA, Franks PW, Hicks JL, Delp SL. Coupled exoskeleton assistance simplifies control and maintains metabolic benefits: A simulation study. PLoS ONE. 2022;17(1):e0261318. 10.1371/journal.pone.0261318. 34986191 10.1371/journal.pone.0261318 PMC8730392

[18] Uchida TK, Seth A, Pouya S, Dembia CL, Hicks JL, Delp SL. Simulating ideal assistive devices to reduce the metabolic cost of running. PLoS ONE. 2016;11(9):e0163417–e0163417. 10.1371/journal.pone.0163417. 10.1371/journal.pone.0163417 PMC5033584 27656901

[19] Luis I, Gutierrez-Farewik EM, ESMAC Best Paper. 2024: Defining exoskeleton aim matters: simulating optimal assistive moments with explicit objectives using bilevel optimization. Gait Posture. 2025;121:315–24. 10.1016/j.gaitpost.2025.06.006. 10.1016/j.gaitpost.2025.06.006 40553916

[20] Neptune RR, Kautz SA, Zajac FE. Contributions of the individual ankle plantar flexors to support, forward progression and swing initiation during walking. J Biomech. 2001;34(11):1387–98. 10.1016/s0021-9290(. 01)00105-1 PubMed PMID: 11672713. 11672713 10.1016/s0021-9290(01)00105-1

[21] Lenhart RL, Francis CA, Lenz AL, Thelen DG. Empirical evaluation of gastrocnemius and soleus function during walking. J Biomech. 2014;47(12):2969–74. 10.1016/j.jbiomech.2014.07.007. 25107666 10.1016/j.jbiomech.2014.07.007 PMC4228932

[22] Hamner SR, Delp SL. Muscle contributions to fore-aft and vertical body mass center accelerations over a range of running speeds. J Biomech. 2013;46(4):780–7. 10.1016/j.jbiomech.2012.11.024 . PubMed PMID: 23246045. 23246045 10.1016/j.jbiomech.2012.11.024 PMC3979434

[23] Delp SL, Loan JP, Hoy MG, Zajac FE, Topp EL, Rosen JM. An interactive graphics-based model of the lower extremity to study orthopaedic surgical procedures. IEEE Trans Biomed Eng. 1990;37(8):757–67. 10.1109/10.102791. 2210784 10.1109/10.102791

[24] Farris DJ, Sawicki GS. Human medial gastrocnemius force-velocity behavior shifts with locomotion speed and gait. Proc Natl Acad Sci USA. 2012;109(3):977–82. 10.1073/pnas.1107972109 . PubMed PMID: 22219360. 22219360 10.1073/pnas.1107972109 PMC3271879

[25] Sawicki GS, Khan NS. A Simple Model to Estimate Plantarflexor Muscle-Tendon Mechanics and Energetics During Walking With Elastic Ankle Exoskeletons. IEEE Trans Biomed Eng. 2016;63(5):914–23. 10.1109/TBME.2015.2491224 . PubMed PMID: 26485350. 26485350 10.1109/TBME.2015.2491224 PMC4874912

[26] Arnold EM, Hamner SR, Seth A, Millard M, Delp SL. How muscle fiber lengths and velocities affect muscle force generation as humans walk and run at different speeds. J Exp Biol. 2013;216(11):2150–60. 10.1242/jeb.075697 . PubMed PMID: 23470656. 23470656 10.1242/jeb.075697 PMC3656509

[27] Pfeifer S, Vallery H, Hardegger M, Riener R, Perreault EJ. Model-based estimation of knee stiffness. IEEE Trans Biomed Eng. 2012;59(9):2604–12. 10.1109. /TBME.2012.2207895 PubMed PMID: 22801482; PubMed Central PMCID: PMC3643895. 22801482 10.1109/TBME.2012.2207895 PMC3643895

[28] Latash ML, Zatsiorsky VM. Joint stiffness: Myth or reality? Hum Mov Sci. 1993;12(6):653–92. 10.1016/0167-9457(93)90010-M.

[29] Roy A, Krebs HI, Patterson SL, Judkins TN, Khanna I, Forrester LW et al. Measurement of human ankle stiffness using the anklebot. In: 2007 IEEE 10th International Conference on Rehabilitation Robotics. 2007. pp. 356–63. https://ieeexplore.ieee.org/abstract/document/442845010.1109/ICORR.2007.4428450. Accessed 17 Apr 2026.

[30] Lee H, Hogan N. Time-varying ankle mechanical impedance during human locomotion. IEEE Trans Neural Syst Rehabil Eng. 2015;23(5):755–64. 10.1109/TNSRE.2014.2346927. 25137730 10.1109/TNSRE.2014.2346927

[31] Rouse EJ, Hargrove LJ, Perreault EJ, Kuiken TA. Estimation of human ankle impedance during walking using the perturberator robot. In: 2012 4th IEEE RAS & EMBS International Conference on Biomedical Robotics and Biomechatronics (BioRob). 2012. pp. 373–8. https://ieeexplore.ieee.org/document/629084210.1109/BioRob.2012.6290842. Accessed 17 Apr 2026.

[32] Sartori M, Maculan M, Pizzolato C, Reggiani M, Farina D. Modeling and simulating the neuromuscular mechanisms regulating ankle and knee joint stiffness during human locomotion. J Neurophysiol. 2015;114(4):2509–27. 10.1152/jn.00989.2014. 26245321 10.1152/jn.00989.2014 PMC4620138

[33] Leboeuf F, Baker R, Barré A, Reay J, Jones R, Sangeux M. The conventional gait model, an open-source implementation that reproduces the past but prepares for the future. Gait Posture. 2019;69:235–41. 10.1016/j.gaitpost.2019.04.015. 31027876 10.1016/j.gaitpost.2019.04.015

[34] Falisse A, Afschrift M, Groote FD. Modeling toes contributes to realistic stance knee mechanics in three-dimensional predictive simulations of walking. PLoS ONE. 2022;17(1):e0256311. 10.1371/journal.pone.0256311. 35077455 10.1371/journal.pone.0256311 PMC8789163

[35] De Groote F, Kinney AL, Rao AV, Fregly BJ. Evaluation of Direct Collocation Optimal Control Problem Formulations for Solving the Muscle Redundancy Problem. Ann Biomed Eng. 2016;44(10):2922–36. 10.1007/s10439-016-1591-9. 27001399 10.1007/s10439-016-1591-9 PMC5043004

[36] Bhargava LJ, Pandy MG, Anderson FC. A phenomenological model for estimating metabolic energy consumption in muscle contraction. J Biomech. 2004;37(1):81–8. 10.1016/S0021-9290. )00239-2 PubMed PMID: 14672571. 14672571 10.1016/s0021-9290(03)00239-2

[37] Uchida TK, Hicks JL, Dembia CL, Delp SL. Stretching your energetic budget: how tendon compliance affects the metabolic cost of running. PLoS ONE. 2016;11(3):e0150378–e0150378. 10.1371/journal.pone.0150378. 10.1371/journal.pone.0150378 PMC4773147 26930416

[38] Galle S, Malcolm P, Collins SH, De Clercq D. Reducing the metabolic cost of walking with an ankle exoskeleton: interaction between actuation timing and power. J Neuroeng Rehabil. 2017;14(1):1–16. 10.1186/s12984-017-0235-0. 28449684 10.1186/s12984-017-0235-0 PMC5408443

[39] Malcolm P, Derave W, Galle S, De Clercq D. A simple exoskeleton that assists plantarflexion can reduce the metabolic cost of human walking. PLoS ONE. 2013;8(2):e56137–e56137. 10.1371/journal.pone.0056137. 10.1371/journal.pone.0056137 PMC3571952 23418524

[40] Poggensee KL, Collins SH. Lower limb biomechanics of fully trained exoskeleton users reveal complex mechanisms behind the reductions in energy cost with human-in-the-loop optimization. Front Robot AI. 2024;11:1283080. 10.3389/frobt.2024.1283080 . PubMed PMID: 38357293; PubMed Central PMCID: PMC10864513. 38357293 10.3389/frobt.2024.1283080 PMC10864513

[41] Franks PW, Bianco NA, Bryan GM, Hicks JL, Delp SL, Collins SH. Testing simulated assistance strategies on a hip-knee-ankle exoskeleton: a case study. In: 2020 8th IEEE RAS/EMBS International Conference for Biomedical Robotics and Biomechatronics (BioRob). New York City, NY, USA: IEEE; 2020. pp. 700–7. https://ieeexplore.ieee.org/document/9224345/10.1109/BioRob49111.2020.9224345. Accessed 4 Oct 2023.

[42] Luis I, Afschrift M, Groote FD, Gutierrez-Farewik EM. Insights into muscle metabolic energetics: Modelling muscle-tendon mechanics and metabolic rates during walking across speeds. PLoS Comput Biol. 2024;20(9):e1012411. 10.1371/journal.pcbi.1012411. 39269982 10.1371/journal.pcbi.1012411 PMC11424009

[43] Shourijeh MS, Fregly BJ. Muscle synergies modify optimization estimates of joint stiffness during walking. J Biomech Eng. 2020;142(1). 10.1115/1.4044310. 10.1115/1.4044310 31343670

[44] Kosterina N, Wang R, Eriksson A, Gutierrez-Farewik EM. Force enhancement and force depression in a modified muscle model used for muscle activation prediction. J Electromyogr Kinesiol. 2013;23(4):759–65. 10.1016/j.jelekin.2013.02.008. 23561824 10.1016/j.jelekin.2013.02.008

[45] Hogan N. Adaptive control of mechanical impedance by coactivation of antagonist muscles. IEEE Trans Autom Control. 1984;29(8):681–90. 10.1109/TAC.1984.1103644.

[46] Cop CP, Schouten AC, Koopman B, Sartori M. Electromyography-driven model-based estimation of ankle torque and stiffness during dynamic joint rotations in perturbed and unperturbed conditions. J Biomech. 2022;145. 10.1016/j.jbiomech.2022.111383. 10.1016/j.jbiomech.2022.111383 36403530

[47] D’Hondt L, Afschrift M, Groote FD. Stochastic optimal control simulations of walking: potential and perspective. bioRxiv; 2026. p. 2026.03.19.712839. https://www.biorxiv.org/content/10.64898/2026.03.19.712839v1. Accessed 22 Apr 2026.

[48] Tessari F, West AM, Hogan N. Explaining human motor coordination via the synergy expansion hypothesis. Proc Natl Acad Sci USA. 2025;122(13):e2501705122. 10.1073/pnas.2501705122. 40146855 10.1073/pnas.2501705122 PMC12002196

[49] Koller JR, Jacobs DA, Ferris DP, Remy CD. Learning to walk with an adaptive gain proportional myoelectric controller for a robotic ankle exoskeleton. J Neuroeng Rehabil. 2015;12(1):97. 10.1186/s12984-015-0086-5. 26536868 10.1186/s12984-015-0086-5 PMC4634144

[50] Mooney LM, Herr HM. Biomechanical walking mechanisms underlying the metabolic reduction caused by an autonomous exoskeleton. J Neuroeng Rehabil. 2016;13:4. 10.1186/s12984-016-0111-3 . PubMed PMID: 26817449; PubMed Central PMCID: PMC4730720. 26817449 10.1186/s12984-016-0111-3 PMC4730720

[51] Falisse A, Serrancolí G, Dembia CL, Gillis J, Jonkers I, De Groote F. Rapid predictive simulations with complex musculoskeletal models suggest that diverse healthy and pathological human gaits can emerge from similar control strategies. J R Soc Interface. 2019;16(157). 10.1098/rsif.2019.0402. 10.1098/rsif.2019.0402 PMC6731507 31431186

[52] Nikoo A, Uchida TK. Be Careful What You Wish for: Cost Function Sensitivity in Predictive Simulations for Assistive Device Design. Symmetry. 2022;14(12):2534. 10.3390/sym14122534.

[53] Ostraich B, Riemer R. Rethinking Exoskeleton Simulation-Based Design: The Effect of Using Different Cost Functions. IEEE Trans Neural Syst Rehabil Eng. 2024;32:2153–64. 10.1109/TNSRE.2024.3409633. 38833397 10.1109/TNSRE.2024.3409633

[54] Weiss A, Chen A, Janischowky DB, Shih I, Phillips GC, Selinger JC et al. Pairing predictive simulations and human experiments in ankle exoskeleton walking. In: 2024 10th IEEE RAS/EMBS International Conference for Biomedical Robotics and Biomechatronics (BioRob). 2024. pp. 925–30. https://ieeexplore.ieee.org/document/1071990510.1109/BioRob60516.2024.10719905. Accessed 21 Apr 2026.

[55] Nguyen VQ, Umberger BR, Sup FC. Predictive simulation of human walking augmented by a powered ankle exoskeleton. In: 2019 IEEE 16th International Conference on Rehabilitation Robotics (ICORR). IEEE; 2019. pp. 53–8. https://ieeexplore.ieee.org/document/8779368/10.1109/ICORR.2019.8779368. 10.1109/ICORR.2019.8779368 31374606

[56] D’Hondt L, Falisse A, Gupta D, Van Den Bosch B, Buurke TJW, Febrer-Nafría M et al. PredSim: a framework for rapid predictive simulations of locomotion. In: 2024 10th IEEE RAS/EMBS International Conference for Biomedical Robotics and Biomechatronics (BioRob). 2024. pp. 1208–13. https://ieeexplore.ieee.org/abstract/document/1071973510.1109/BioRob60516.2024.10719735. Accessed 11 Feb 2025.

[57] Collins SH, Groote FD, Gregg RD, Huang H (Helen), Lenzi T, Sartori M Experiment-free learning of exoskeleton assistance remains an unsolved problem. bioRxiv; 2026. p. 2026.04.01.715109. https://www.biorxiv.org/content/10.64898/2026.04.01.715109v1. Accessed 18 Jun 2026.

[58] Canete S, Wilson EB, Jacobs DA. Ankle Exoskeleton Assistance Can Affect Step Regulation During Self-Paced Walking. IEEE Trans Neural Syst Rehabil Eng. 2023;31:474–83. 10.1109/TNSRE.2022.3226766. 37015569 10.1109/TNSRE.2022.3226766

