# Supplementary material for: Why inverse simulations overestimate optimal ankle exoskeleton assistance: the role of bi-articular coordination and joint stiffness
Source: J Neuroeng Rehabil. 2026 Jul 21;23:224. doi: 10.1186/s12984-026-02106-3 (PMC13386900; doi:10.1186/s12984-026-02106-3)
Supplement: Supplementary file 1 — Supplementary Material 1 [file 12984_2026_2106_MOESM1_ESM.docx]

**Supplementary Material**


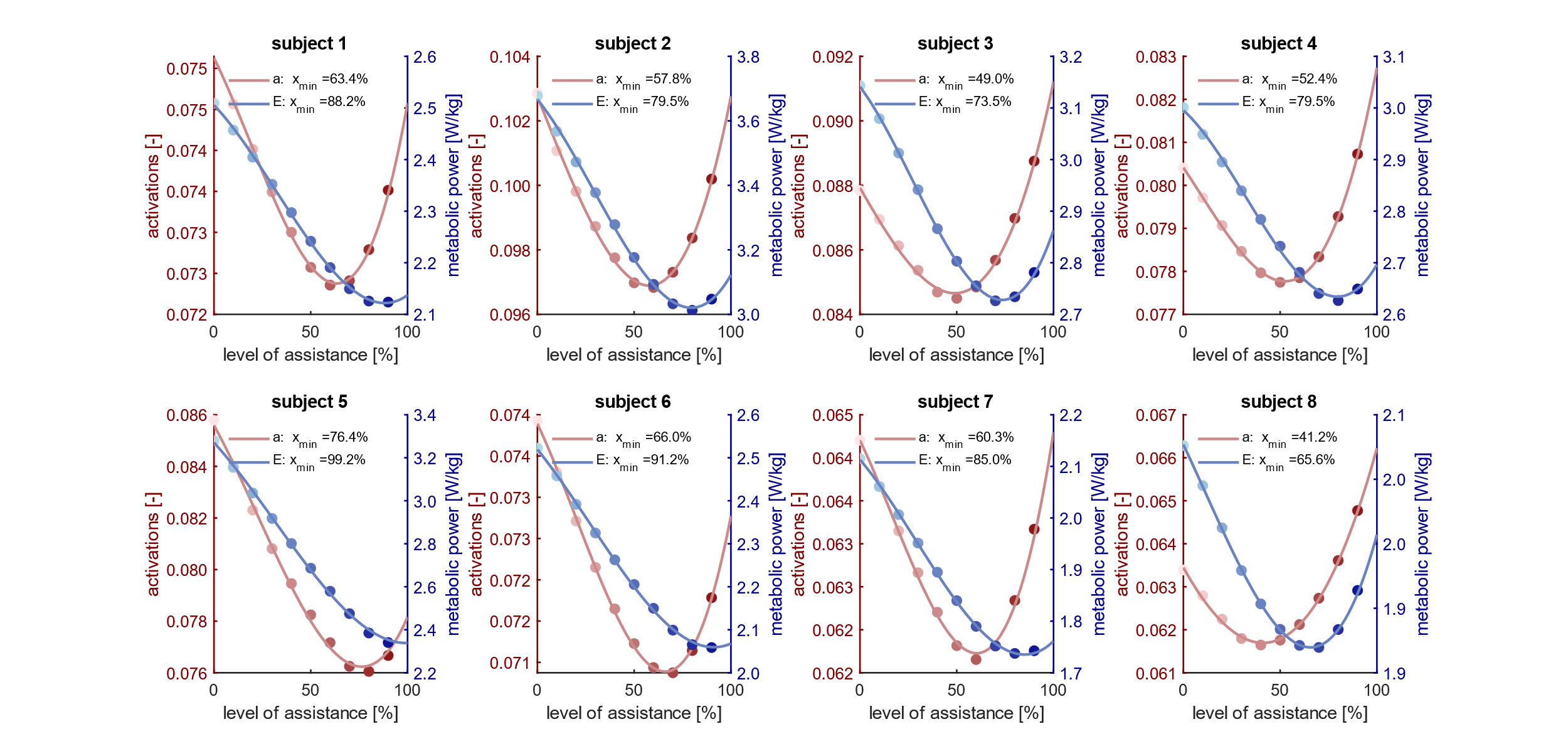


*Supplementary Figure 1: Mean muscle activations and metabolic power across levels of assistance for each subject.*


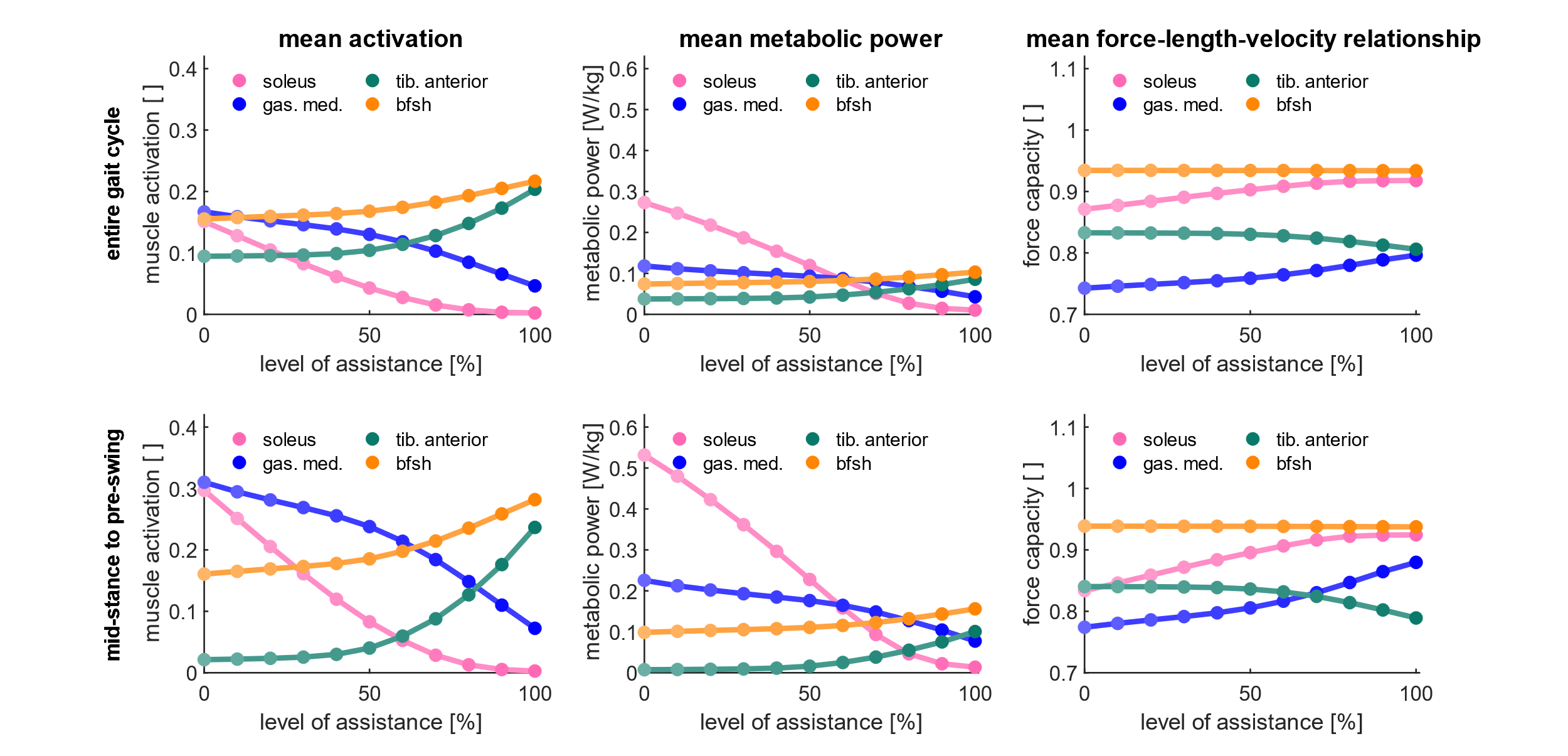


*Supplementary Figure 2: Mean muscle activations, metabolic rates, and force-length-velocity relationship of soleus, gastrocnemius medialis, tibialis anterior, and biceps femoris short head (bfsh) muscles across levels of assistance. Mean values were computed with respect to the entire gait cycle (upper row) and mid-stance to pre-swing phase, 12 to 62% of the gait cycle. The force-length-velocity relationship represents the active force generation capacity of the muscle, computed as the product of the force-length and force-velocity relationship. Average values were computed among all subjects.*
